# Palliative Care in Advanced Head and Neck Cancer: Multidisciplinary Management and End-of-Life Decision-Making

**DOI:** 10.7759/cureus.109081

**Published:** 2026-05-18

**Authors:** Vanessa Duarte Branco, Catarina Dias, Inês Rento, Débora Cardoso, Ana Luísa Esteves

**Affiliations:** 1 Oncology, Unidade Local de Saúde de Lisboa Ocidental (ULS Lisboa Ocidental), Hospital de São Francisco Xavier, Lisboa, PRT; 2 Oncology, Unidade Local de Saúde do Alto Ave (ULS Alto Ave), Hospital de Guimarães, Guimarães, PRT; 3 Internal Medicine, Unidade Local de Saúde de Viseu Dão-Lafões (ULS Viseu Dão-Lafões), Hospital de São Teotónio, Viseu, PRT; 4 Internal Medicine, Hospital da Luz, Lisbon, PRT

**Keywords:** advance palliative care planning, end-of-life care, head and neck cancer, multidisciplinary palliative care, palliative care, symptom management

## Abstract

Head and neck cancers (HNCs) represent a heterogeneous group of malignancies with high morbidity and complex functional impairment. Despite advances in multimodal therapy, many patients present with advanced or incurable disease, demanding early integration of palliative care.

To review the epidemiology, symptom burden, and best practices in palliative care for patients with advanced HNC, highlighting strategies for symptom management, communication, and end-of-life decision-making.

A narrative review of current literature, clinical guidelines (National Comprehensive Cancer Network (NCCN), American Society of Clinical Oncology (ASCO), UK National Multidisciplinary Guidelines), and key studies on palliative interventions in HNC was conducted. Emphasis was placed on early integration of palliative care, multidisciplinary management, and evidence-based approaches to symptom control.

HNC patients experience high symptom burden, including pain, dysphagia, airway compromise, hemorrhage, and psychological distress. Early palliative care integration improves symptom control, quality of life, and alignment of treatment with patient goals. Multidisciplinary approaches involving surgeons, oncologists, palliative specialists, speech therapists, dietitians, and psychosocial support are essential. Sentinel clinical events should trigger structured discussions on prognosis, treatment objectives, and end-of-life preferences. Advance care planning, including documentation of "do not resuscitate" (DNR) orders and preferred location of death, is critical in the terminal phase.

Effective palliative care in HNC requires early, proactive, and multidisciplinary strategies that balance disease-directed therapy with patient-centered goals. Standardization of care pathways and ongoing evaluation of emerging evidence are essential to optimize symptom management, facilitate informed decision-making, and enhance quality of life for patients with advanced diseases.

## Introduction and background

The Global Cancer Observatory describes head and neck cancers (HNC) as a diverse group of malignancies and ranks them among the most common cancers globally, with an estimated 900,000 new cases and more than 450,000 deaths each year [1]. Most tumors are squamous cell carcinomas arising from the mucosal lining of the oral cavity, oropharynx, larynx, and hypopharynx [1,2]. Historically, tobacco exposure and alcohol consumption have been the predominant risk factors, with a well-established synergistic effect on carcinogenesis [2].

Over recent decades, a marked epidemiological shift has been observed, largely driven by the increasing incidence of human papillomavirus (HPV)-associated oropharyngeal cancer [3]. In many high-income countries, HPV-positive disease now represents the majority of oropharyngeal tumors and is characterized by distinct clinical and biological features, including a more favorable prognosis compared with HPV-negative cancers [3,4].

In the United States, approximately 70,000 new cases of HNC are diagnosed annually, with higher incidence rates in men and a median age at diagnosis in the mid-60s [5]. Data from population-based registries such as the Surveillance, Epidemiology, and End Results (SEER) Program highlight persistent disparities in both incidence and outcomes, influenced by demographic and socioeconomic factors [5]. Despite therapeutic advances, a significant proportion of patients continue to present with advanced-stage disease, contributing to substantial morbidity and mortality [1-5].

## Review

Objectives and methods

This manuscript aims to examine the role of palliative care in advanced HNC, with particular emphasis on symptom burden, multidisciplinary management, and end-of-life care strategies. The objective is to provide a comprehensive overview of current evidence-based approaches to symptom control, communication, advance care planning, and the management of critical complications such as airway obstruction and catastrophic hemorrhage.

The methodology consisted of a structured narrative review of the literature. Searches were conducted in PubMed and relevant guideline repositories between March and May 2026, including publications from 2015 to 2026. Keywords used included “head and neck cancer,” “palliative care,” “symptom management,” “end-of-life care,” “palliative sedation,” and “catastrophic hemorrhage.” Articles published in English were selected based on their relevance to palliative care integration, multidisciplinary management, and clinical outcomes in advanced disease. International guidelines, including those from the National Comprehensive Cancer Network (NCCN), American Society of Clinical Oncology (ASCO), and the United Kingdom National Multidisciplinary Guidelines, were also reviewed to support evidence-based clinical recommendations.

HNC: palliative care

HNC encompasses a heterogeneous group of malignant neoplasms characterized by substantial morbidity, complex treatment pathways, and significant functional impairment [6]. Dunn et al. highlighted that, although advances in multimodal therapy have improved outcomes in selected populations, a considerable proportion of patients continue to present with locally advanced or incurable disease, with median survival remaining limited in recurrent or metastatic settings [6]. These clinical realities demand careful consideration of treatment intent and underscore the importance of shared decision-making throughout the disease trajectory, particularly when the probability of cure is low [6].

Beyond oncologic outcomes, HNC is uniquely associated with profound effects on essential functions, including speech, swallowing, respiration, and appearance. These factors contribute to a high symptom burden and complex psychosocial distress, making the advanced disease trajectory particularly challenging for both patients and clinicians [7]. White et al. reported that advanced disease is frequently characterized by progressive functional deterioration, airway compromise, dysphagia, hemorrhage, and pain, often requiring prompt and coordinated supportive and palliative interventions [7].

In a study carried out by Merikari et al., early palliative care decisions were associated with reduced hospitalizations, fewer emergency department visits, and a lower likelihood of aggressive interventions in the final stages of life [8]. Despite these benefits, the integration of palliative care in head and neck oncology remains inconsistent, with only 33.1% of patients having contact with a specialist palliative care unit before death and only 23.8% receiving early specialist palliative care contact [8]. As a result, end-of-life care is frequently characterized by high healthcare utilization and potentially burdensome interventions. Emerging evidence suggests that the timing of palliative care decision-making, particularly the transition from life-prolonging treatments to comfort-focused care, plays a critical role in the quality of end-of-life care [8].

Palliative care should be introduced early in the course of advanced NHC, ideally alongside active disease-directed treatment from the time of advanced disease diagnosis, rather than being reserved for the terminal phase. Importantly, early palliative care should not be equated with end-of-life care. Its role is to support symptom control, communication, nutritional and psychosocial needs, treatment decision-making, and advance care planning throughout the disease trajectory [9-11].

The presence of symptoms such as pain, dysphagia, mucositis, airway discomfort, xerostomia, or psychological distress during active treatment does not, in itself, indicate a transition to end-of-life care, as many of these symptoms may improve with disease-directed therapy. Instead, the need for end-of-life discussions should be guided by the overall disease trajectory, response to treatment, functional decline, prognosis, treatment tolerance, and patient preferences [9-11].

A key component of early palliative care is timely and structured communication regarding prognosis and treatment objectives, ideally initiated early after the diagnosis of advanced or incurable disease. These discussions should include identification and documentation of a legally authorized decision-maker, establishment of advance directives, and comprehensive exploration of patient values, preferences, and priorities. Treatment goals should not be viewed as static, but rather dynamic and evolving, requiring regular reassessment as the disease progresses and clinical circumstances change. Continuous dialogue among patients, caregivers, and the multidisciplinary team is essential to ensure that treatment decisions remain consistent with patient preferences and focused on maintaining quality of life [9-11].

Current international guidelines recommend that palliative care be incorporated as a standard component of multidisciplinary management. The NCCN emphasizes that all patients with HNC should have access to palliative care services throughout the disease trajectory, whereas the ASCO advocates early referral to specialized palliative care teams, ideally within the first eight to 12 weeks following a diagnosis of advanced cancer [9,10].

Referral to specialized palliative care is particularly indicated for patients with advanced or metastatic disease, uncontrolled symptoms including refractory pain, severe dysphagia, xerostomia, poor functional status, or complex psychosocial needs [10]. Additionally, frequent hospitalizations, emergency department visits, or the need for discussions regarding treatment goals should prompt early involvement of palliative care teams [10].

Given the complexity of HNC, optimal care requires a multidisciplinary approach involving head and neck surgeons, medical and radiation oncologists, palliative care specialists, speech and language therapists, dietitians, and psychosocial support services [11].

Collectively, these findings underscore that decision-making in advanced HNC extends beyond purely clinical considerations and represents a critical component of high-quality care. The complex morbidity associated with the disease, coupled with prognostic uncertainty and the profound impact of treatment on essential human functions, renders this population particularly vulnerable to both inadequate symptom management and potentially burdensome interventions near the end of life. Consequently, optimizing both the timing and content of palliative care discussions is crucial to ensure that care remains consistently aligned with patients’ values, goals, and priorities [9-11].

Symptom management in HNC

Patients with advanced HNC experience a complex and often severe symptom burden resulting from both tumor progression and treatment-related toxicity. The anatomical location of the disease, involving structures essential for respiration, swallowing, and communication, creates a unique clinical scenario in which multiple distressing symptoms frequently coexist [7,12]. Effective management requires a proactive, multidisciplinary approach that balances symptom control with treatment burden while aligning care with patient goals [7].

Pain Management

Pain is one of the most prevalent and debilitating symptoms in HNC, arising from tumor infiltration, inflammation, mucosal injury, and neuropathic mechanisms due to cranial nerve involvement [12,13]. Management should follow a stepwise approach based on the World Health Organization (WHO) analgesic ladder, incorporating both non-opioid and opioid therapies as appropriate [13].

Neuropathic pain components are common and may require adjuvant agents such as gabapentin, pregabalin, or tricyclic antidepressants [12]. In refractory cases, early referral to specialized palliative care or pain management services is recommended, and interventional techniques, including nerve blocks, may be considered [12]. Although opioid therapy remains central to effective pain control, its use must be carefully balanced against potential adverse effects, including sedation and respiratory compromise, particularly in patients with airway involvement [12].

Airway Obstruction

Airway obstruction is a critical and potentially life-threatening complication in advanced HNC, often resulting from tumor progression, edema, hemorrhage, or secretion retention [7,12]. Patients may present with dyspnea, stridor, and significant anxiety, requiring prompt assessment and intervention.

Treatment options include corticosteroids to reduce inflammatory edema, palliative radiotherapy to decrease tumor burden, and surgical airway interventions such as tracheostomy [12]. Each approach has specific benefits and limitations: tracheostomy can provide immediate airway relief but may adversely affect communication and quality of life, whereas radiotherapy can achieve tumor control but has a delayed effect and potential toxicity [7,12].

In terminally ill patients, less invasive measures, including opioids for dyspnea and secretion management, may be preferable, with palliative sedation considered in refractory cases [7].

Dysphagia and Nutritional Support

Dysphagia is highly prevalent in patients with advanced HNC and contributes substantially to malnutrition, aspiration, and reduced quality of life [12,13]. It may result from tumor obstruction, neuromuscular dysfunction, or treatment-related fibrosis and xerostomia. According to Weaver et al., early engagement of speech and language therapists and dietitians is crucial to optimize nutritional intake and maintain swallowing function [12]. Management strategies include texture-modified diets, swallowing rehabilitation, and enteral feeding via nasogastric or gastrostomy tubes [12,14].

Gastrostomy provides reliable nutritional support for selected patients but may be associated with complications and reduced oral intake. When safe, maintenance of oral intake is encouraged to preserve function and quality of life [12,14]. Decisions regarding nutritional interventions should be individualized and aligned with overall care goals, particularly in the palliative context [7].

Hemorrhage

Hemorrhage is a serious and feared complication in advanced HNC, ranging from minor bleeding to catastrophic events such as carotid artery rupture. Risk factors include tumor invasion of vascular structures, prior radiotherapy, and infection [12].

Management strategies encompass local hemostatic measures, antifibrinolytic agents such as tranexamic acid, palliative radiotherapy, and endovascular interventions, including embolization [12]. The efficacy of these approaches depends on the severity and source of bleeding [12].

In cases of catastrophic hemorrhage, management focuses on rapid symptom control and patient comfort. As outlined in the United Kingdom National Multidisciplinary Guidelines, catastrophic hemorrhage in patients with head and neck malignancies is typically sudden and frequently terminal, with minimal opportunity for definitive intervention [11]. Management should therefore prioritize rapid alleviation of distress, including administration of fast-acting sedative agents, particularly benzodiazepines, to mitigate severe anxiety and agitation. Although not always formally described as palliative sedation, this approach is consistent with the principles of proportionate sedation in an emergency setting, with the primary goal of symptom control. The guideline further highlights the importance of anticipatory planning and maintaining continuous presence at the patient’s side as key measures to preserve comfort and dignity [11]. Advance discussions and planning are essential to minimize distress for both patients and caregivers [12].

Psychological and social impact

The psychological impact of HNC is substantial, driven by functional impairment, visible disfigurement, communication difficulties, and consequent social isolation [7,12]. Patients experience higher rates of depression, anxiety, and suicide compared with other cancer populations [6].

Loss of speech and changes in appearance can significantly affect identity and interpersonal relationships, while fear of airway compromise or hemorrhage further contributes to distress [12]. Management requires routine psychological screening, early involvement of mental health professionals, and comprehensive psychosocial support. Effective communication and advance care planning are fundamental, facilitating informed decision-making and alignment of treatment with patient values and preferences [6,7].

Integrated approach and communication

Palliative management of advanced HNC demands continuous reassessment of symptom burden, treatment efficacy, and patient priorities. The interplay between physical symptoms and psychological distress requires a holistic, multidisciplinary approach [12].

Effective Communication and Transition Points

Effective communication between patients and healthcare professionals is essential for the timely integration of palliative care and for identifying critical transition points throughout the disease trajectory. The ASCO consensus guideline emphasizes that specific clinical and situational “sentinel events” should trigger structured discussions regarding prognosis, care objectives, and treatment preferences [15]. These events include the diagnosis of advanced or incurable disease, disease progression despite therapy, functional decline, recurrent hospitalizations, and development of substantial symptom burden, particularly in patients who are bedbound, semi-comatose, able to take only minimal oral intake such as small sips of fluids, or unable to tolerate oral medications [15,16].

Gilligan et al. emphasize that situational sentinel events provide critical opportunities to reassess care goals and to initiate or escalate involvement of palliative care services [15]. In advanced HNC, these events should not be interpreted as isolated symptoms alone, but rather as clinical turning points that signal a need for structured reassessment of prognosis, treatment goals, and patient preferences. Relevant triggers may include disease progression despite active treatment, recurrent unplanned hospital admissions, repeated emergency department visits, progressive functional decline, declining performance status, treatment intolerance, airway compromise, severe dysphagia with nutritional failure, major bleeding or sentinel hemorrhage, and increasing dependence on supportive interventions [11,15,16].

Recognition of these sentinel events should prompt multidisciplinary discussion and early palliative care involvement. However, the transition to comfort-focused or end-of-life care should be individualized and should depend on prognosis, treatment response, performance status, expected benefits and burdens of further oncologic therapy, and the patient’s goals of care [15-17]. This distinction is essential because symptoms such as pain, dysphagia, mucositis, or airway discomfort may occur during active treatment and may improve with disease-directed therapy. They should therefore not automatically be interpreted as indicators of terminal decline or as reasons to discontinue active treatment [11,15-17].

A critical aspect of care in advanced HNC is identifying when curative-intent or disease-directed therapy is no longer beneficial and may instead contribute to patient suffering. This transition is often difficult to define, as disease-directed and palliative therapies frequently coexist, and interventions such as chemotherapy or radiotherapy may be administered with either curative or palliative intent depending on the clinical context [17].

Continuation of antineoplastic therapy in the absence of a realistic prospect of benefit may lead to unnecessary physical and psychological harm, prolonging life without meaningful improvement in quality. Timely recognition of this threshold is essential to avoid futile interventions and to facilitate transition to a palliative care-focused approach when appropriate [17]. This process requires shared decision-making and honest communication regarding prognosis, treatment goals, and patient preferences [17].

End-of-life considerations in HNC

Palliative care for patients with HNC requires careful evaluation of interventions that may no longer provide meaningful benefit and may instead contribute to patient suffering. Artificial hydration and nutrition, although often perceived as supportive measures, can become futile during the terminal phase and may lead to complications such as peripheral edema, nausea, and pulmonary congestion. Decisions regarding continuation should be guided by patient goals, symptom burden, and overall prognosis [11,16].

Certain complications unique to HNC, such as carotid blowout syndrome, represent rare but catastrophic events that require advance planning. Patients identified as high risk may require hospitalization, and home caregivers should be prepared with practical measures, including the availability of dark towels to minimize visual distress in the event of severe bleeding [11,16].

Advance care planning is a critical component of high-quality palliative care. Do-not-resuscitate (DNR) orders should be proactively discussed and clearly documented, particularly for patients receiving care at home. Additionally, clinicians should ascertain and document the patient’s preferred location of death and take measures to facilitate this whenever feasible. Referral to specialized palliative care services should be considered for patients with an anticipated life expectancy of less than six months, enabling transition to comfort-focused care and support for both patients and families [11,16].

## Conclusions

Effective palliative care in HNC requires early, proactive, and multidisciplinary integration alongside disease-directed treatment. Early palliative care should not be equated with end-of-life care; rather, it supports symptom control, communication, nutritional and psychosocial needs, advance care planning, and alignment of treatment with patient goals throughout the disease trajectory. This distinction is particularly important in HNC, where symptoms such as pain, dysphagia, airway discomfort, mucositis, and psychological distress may occur during active treatment and may improve with appropriate oncologic and supportive interventions.

Clear recognition of sentinel clinical events can guide timely reassessment of prognosis, treatment objectives, and preferences for care. Disease progression despite therapy, recurrent unplanned hospitalizations, functional decline, treatment intolerance, airway compromise, nutritional failure, major bleeding, and escalating dependence on supportive interventions should prompt structured multidisciplinary discussion and early palliative care involvement. Standardized care pathways and continued evaluation of emerging evidence are essential to optimize symptom management, support informed decision-making, and improve quality of life for patients with advanced HNC.
